# Prophylaxis of Preeclampsia With Low-Dose Aspirin: A Comprehensive Review

**DOI:** 10.7759/cureus.96529

**Published:** 2025-11-10

**Authors:** Hira Sohail

**Affiliations:** 1 Obstetrics and Gynaecology, Tongji University, Shanghai, CHN

**Keywords:** foetal growth restriction (fgr) preeclampsia, hypertension in pregnancy, low dose aspirin, preeclampsia, pre-eclampsia prophylaxis

## Abstract

Preeclampsia (PE) is a multifactorial hypertensive disorder of pregnancy and a leading cause of maternal and perinatal morbidity and mortality worldwide. Despite decades of research, its precise pathophysiology remains incompletely understood, though abnormal placentation, endothelial dysfunction, angiogenic imbalance, oxidative stress, and immune dysregulation are recognized contributors. Low-dose aspirin (LDA) has emerged as a safe and effective prophylactic intervention in high-risk pregnancies. It acts primarily by irreversibly inhibiting cyclooxygenase enzymes, restoring the thromboxane A₂/prostacyclin balance, reducing platelet aggregation, and improving uteroplacental perfusion. The daily doses between 60 and 150 mg - most commonly 75 mg - initiated before 16 weeks of gestation significantly reduce the incidence of preterm and early-onset PE, as well as associated complications such as fetal growth restriction (FGR) and preterm delivery. Large clinical trials and meta-analyses have demonstrated that LDA is well tolerated, with minimal risk of maternal or neonatal bleeding. However, challenges persist regarding optimal dose standardization, adherence, and implementation in low-resource settings. Ongoing research emphasizes the need for biomarker-guided screening, personalized dosing, and early risk stratification to enhance prophylactic outcomes. Overall, LDA represents a cost-effective, evidence-based, and globally accessible intervention that substantially reduces the burden of hypertensive disorders in pregnancy and improves maternal-fetal outcomes when administered in an appropriate and timely manner.

## Introduction and background

Preeclampsia (PE) is a pregnancy complication that occurs in about 3-8% of women and continues to be one of the main causes of both maternal and neonatal illness and death, affecting health outcomes in the short and long term [1]. It is characterized by hypertension and proteinuria, and if left untreated, can result in significant risks to both maternal and fetal health [2]. It has also been strongly associated with various complications that can result in pregnancy loss and increased inflammation [3]. Although extensive research has been conducted, the precise etiology of PE is still unclear. However, factors such as abnormal placentation, immune system dysregulation, and endothelial dysfunction are thought to play key roles in its development [4]. Adverse outcomes linked to PE include hypertension, proteinuria, placental abnormalities, and fetal growth restriction [2].

The primary treatment for PE is the delivery of the baby, which often results in preterm birth and poses additional risks for both the mother and the child [5]. Hence, researchers have shown considerable interest in developing preventive strategies aimed at reducing the risk of PE in high-risk pregnancies. A well-known intervention is the use of low-dose aspirin (LDA), a treatment that is known to improve vascular function, along with reducing the risk of developing PE. This intervention is particularly recommended for women with certain risk factors, including a previous history of PE, chronic hypertension, or multiple pregnancies [1,2,6,7]. At 11-13 weeks of gestation, screening for PE using maternal factors such as demographic data, medical history, and biomarker levels can detect approximately 75% of women who will develop preterm PE (delivery before 37 weeks) and around 90% of those who will develop early-onset PE (delivery before 32 weeks), with a 10% false-positive rate [7].

LDA exerts its therapeutic effect by inhibiting platelet aggregation through the suppression of thromboxane A2 synthesis [8,9]. This mechanism promotes optimal uteroplacental blood flow and decreases the likelihood of placental insufficiency, a hallmark of PE. Recent evidence indicates that the early initiation of LDA during pregnancy - typically between 12 and 16 weeks of gestation - can substantially reduce the incidence of PE and improve perinatal outcomes by minimizing the risks of fetal growth restriction (FGR) and preterm birth [10]. A more comprehensive understanding of the underlying pathophysiological mechanisms may enhance early detection of these complications, although this remains a significant clinical challenge. During pregnancy, the maternal cardiovascular system undergoes dynamic and continuous adaptations, some of which are modulated by the autonomic nervous system [11].

## Review

Methodology

This comprehensive review was conducted to evaluate the role of LDA in the prevention of PE. A structured literature search was performed using PubMed, ScienceDirect, and the American Journal of Obstetrics and Gynecology databases. The search utilized combinations of the keywords “preeclampsia,” “low-dose aspirin,” “aspirin prophylaxis,” “pregnancy hypertension,” and “fetal growth restriction.” Articles published in English between 2000 and 2025 were included. Eligible studies comprised clinical trials, observational studies, systematic reviews, and meta-analyses that investigated the preventive or therapeutic effects of LDA in pregnancy. Publications not involving human subjects, non-peer-reviewed material, conference abstracts, and case reports were excluded.

The selected studies were analyzed thematically to explore the mechanisms of action of LDA, optimal dosage and timing, safety considerations, and clinical outcomes in the prevention of PE. The findings were synthesized narratively to provide a comprehensive overview of current evidence and clinical practice recommendations.

Pathophysiology of preeclampsia

The exact pathophysiology of PE is still not fully understood, nor is the precise mechanism by which LDA prevents the condition [8,12,13]. Its preventive effect is believed to result from aspirin’s anti-inflammatory and anti-platelet actions [13]. When taken before 16 weeks of gestation, LDA reduces platelet aggregation and promotes vasodilation, improving uteroplacental blood flow [14]. Various pharmacologic agents, such as low-molecular-weight heparin, antioxidants, calcium, proton-pump inhibitors, metformin, and statins, have been explored for the prevention and management of PE; however, LDA has shown the most consistent efficacy [15]. Although there have been concerns regarding potential adverse effects of non-steroidal anti-inflammatory drugs - such as maternal or fetal bleeding and premature closure of the fetal ductus arteriosus - large clinical studies show that LDA is generally safe in pregnancy, posing minimal risk. A prior history of PE raises the recurrence risk to approximately 25%, while chronic hypertension accounts for 15-20%. Multiple pregnancies and advanced maternal age contribute 10-15% of cases, and obesity has a prevalence rate of 25-30%, making it a strong risk factor [16]. These statistics underscore the importance of early screening and preventive measures.

Abnormal Placentation and Vascular Remodeling

In a normal pregnancy, trophoblastic cells invade the maternal spiral arteries, transforming them into dilated, low-resistance vessels that ensure an adequate supply of oxygen and nutrients to the fetus. In PE, this remodeling is inadequate or superficial, leading to high-resistance blood flow and episodes of placental hypoxia. The underperfused placenta then releases various bioactive substances into the maternal bloodstream, causing widespread vascular dysfunction [17].

Angiogenic Imbalance and Endothelial Damage

One of the most characteristic features of preeclampsia is the disruption of angiogenic homeostasis. Normally, vascular endothelial growth factor (VEGF) and placental growth factor (PlGF) support vascular health and placental perfusion. In preeclamptic pregnancies, these proangiogenic mediators are suppressed by excessive antiangiogenic molecules such as soluble fms-like tyrosine kinase-1 (sFlt-1) and soluble endoglin. The resulting reduction in nitric oxide production and endothelial integrity promotes vasoconstriction, fluid leakage, and hypertension [18].

Oxidative Stress and Inflammatory Response

Insufficient placental perfusion leads to the generation of reactive oxygen species (ROS), producing oxidative stress within both the placenta and maternal tissues. This oxidative burden damages lipids and proteins, stimulates inflammatory cytokines like interleukin-6 (IL-6) and tumor necrosis factor-alpha (TNF-α), and contributes to endothelial injury. Leukocyte activation and complement overactivity further exacerbate the inflammatory environment, intensifying vascular dysfunction [19,20].

Immune Dysregulation and Genetic Predisposition

Maternal immune tolerance to fetal antigens is crucial for normal placental development. In preeclampsia, immune maladaptation - such as an imbalance between pro-inflammatory and regulatory immune cells - can disrupt trophoblastic invasion. Genetic variations that affect oxidative stress regulation, angiogenic signaling, or immune function may also increase susceptibility to the disorder. In addition, certain microRNAs have been implicated in altering gene expression patterns that control placental invasion and vascular remodeling [21].

Systemic Manifestations and Organ Injury

The maternal clinical features of preeclampsia - hypertension, proteinuria, and multi-organ impairment - stem from generalized endothelial injury and vasospasm. Reduced blood flow to key organs such as the kidneys, liver, and brain leads to renal insufficiency, hepatic enzyme elevation, neurological disturbances, and, in severe cases, eclamptic seizures. Placental ischemia also contributes to fetal growth restriction and adverse perinatal outcomes [22].

Aspirin for preeclampsia prevention

One factor that may affect the success of PE prevention is the aspirin dose used. LDA, when initiated at the end of the first trimester after organogenesis is complete, has not been associated with fetal abnormalities [23]. Differences in drug response among populations, regions, and ethnic groups can arise from a mix of genetic, metabolic, and pharmacokinetic factors, as well as environmental and dietary influences [24]. Determining the appropriate aspirin dose is essential to maximize both safety and effectiveness, with the main goal being to maintain a balance between thromboxane and prostacyclin activity [25].

The 2019 NICE guidelines recommend a daily dose of 75 mg of LDA, which is sufficient to achieve near-complete inhibition of cyclooxygenase-1 (COX-1) and suppress thromboxane A₂ (TXA₂) production in most pregnant women. Clinical evidence shows that doses between 60 mg and 150 mg per day effectively reduce TXA₂ activity and lower the risk of preeclampsia, while higher doses do not provide additional benefit [26]. Doses above this range may also suppress prostacyclin synthesis, potentially disrupting vascular balance. For these reasons, 75 mg daily remains the most commonly recommended and well-tolerated prophylactic regimen [27].

Timing of administration of low-dose aspirin

Early identification of women at risk for preeclampsia is essential. Guidelines from the American College of Obstetricians and Gynecologists (ACOG) and the US Preventive Services Task Force (USPSTF) recommend starting LDA during the second trimester [28,29]. However, evidence indicates that initiating aspirin after 16 weeks of gestation may reduce its preventive effectiveness. For example, a study in China found that daily administration of 100 mg aspirin from 12 to 20 weeks until 34 weeks did not significantly lower the incidence of preeclampsia in high-risk women [25].

**Figure 1:**
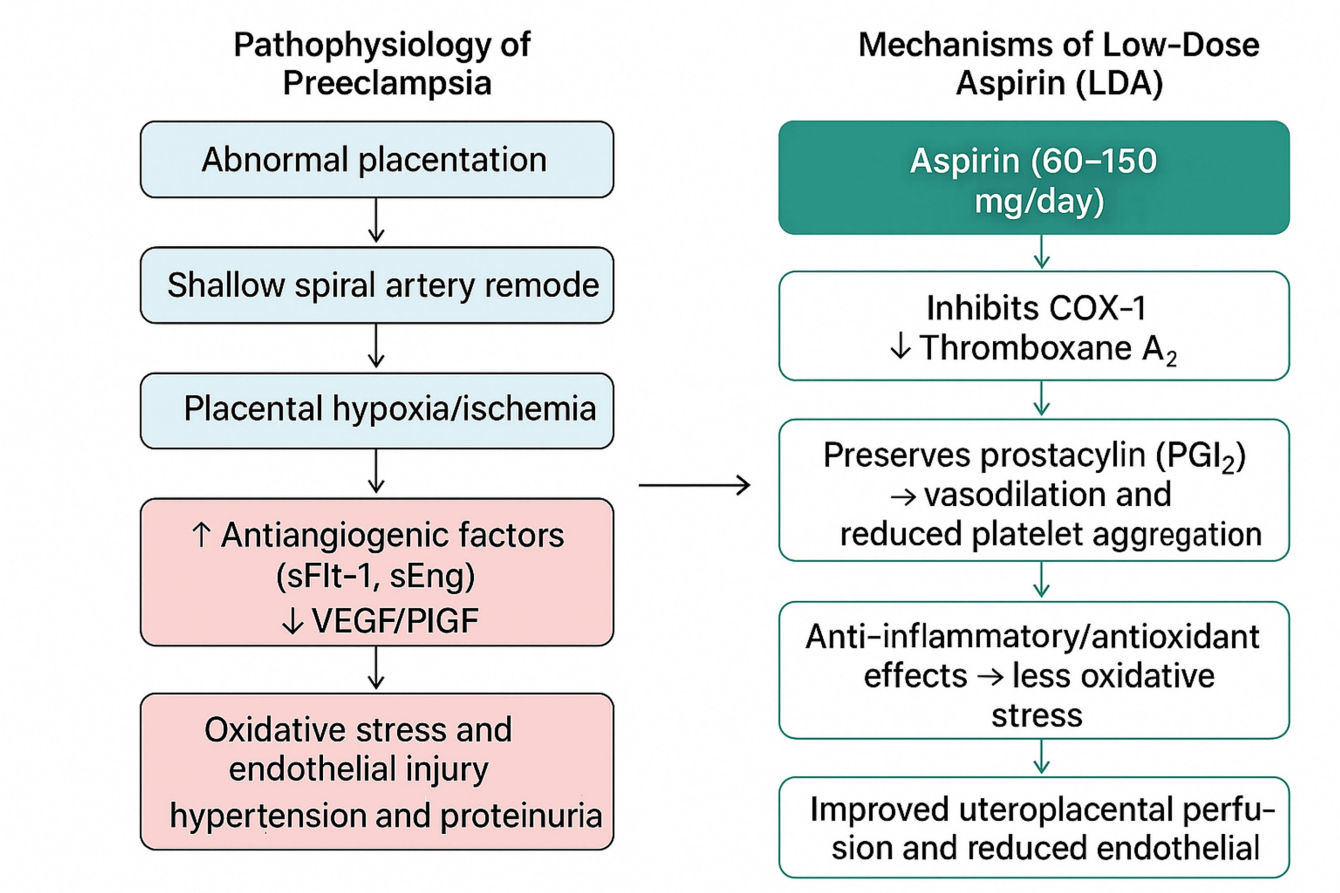
Schematic overview of the pathophysiology of preeclampsia and mechanisms of low-dose aspirin* ^*^Adapted from references [13,14,17,18,19,27] Abnormal placentation leads to placental ischemia and hypoxia, resulting in elevated antiangiogenic factors (sFlt-1, sEng) and decreased VEGF/PlGF, which contribute to oxidative stress, endothelial dysfunction, and systemic vasoconstriction. Low-dose aspirin inhibits COX-1, reducing thromboxane A₂ synthesis while preserving prostacyclin-mediated vasodilation, thereby improving uteroplacental blood flow and reducing inflammation and oxidative stress Figure created by the author using artificial intelligence tools and Canva VEGF: vascular endothelial growth factor; PlGF: placental growth factor

A thorough evaluation of personal and family medical history is crucial for identifying those most likely to benefit from LDA therapy. Recognizing risk factors early enables timely discussions about the suitability of prophylaxis and the optimal timing for initiation [10]. According to the 2022 USPSTF guidelines, routine LDA is not advised for primary prevention in women under 50 or over 70 years with cardiovascular disease. Women aged 50-59 years with a 10-year cardiovascular risk above 10% receive a Grade B recommendation for aspirin use, while those aged 60-69 years receive a Grade C recommendation, highlighting the need for individualized decisions due to increased bleeding risk with age. Figure 1 illustrates the proposed pathophysiological mechanisms of preeclampsia and the multifaceted role of LDA in modulating endothelial dysfunction, platelet aggregation, and placental perfusion.

Adverse effects and safety considerations

For women diagnosed with preeclampsia or gestational hypertension with severe features at 34 weeks’ gestation or later, delivery is recommended once the mother’s condition is stabilized, due to the potential risks to both mother and infant [30]. Women with chronic hypertension often exhibit pre-existing endothelial dysfunction and inflammatory markers, making them more susceptible to placental injury and subsequent development of preeclampsia [31]. Steroid administration should not delay delivery. In cases occurring before 34 weeks, expectant management may be considered through shared decision-making, taking into account local resources and maternal-fetal status. Delivery should proceed immediately if maternal or fetal conditions deteriorate, as continuing pregnancy may increase maternal risk without providing benefit to the infant [32].

Severe hypertension is defined as persistent elevated blood pressure lasting at least 15 minutes, and should be managed promptly, with antihypertensive therapy initiated within 60 minutes [33]. Common treatments include oral nifedipine or intravenous hydralazine, or labetalol. It is to avoid such adverse outcomes as myocardial ischemia, congestive heart failure, renal failure, and stroke. Maternal and neonatal bleeding are rare and categorized as low-risk events [34]. Antenatal closure of the ductus arteriosus is also rare but carries a moderate risk level. Gastrointestinal issues occur occasionally but are generally low risk [35]. These findings support the overall safety of LDA in high-risk pregnancies.

Global epidemiology and risk factors

Preeclampsia remains a major global health concern, affecting maternal and neonatal outcomes across all regions of the world [36]. Its prevalence varies significantly depending on geographic location, socioeconomic conditions, and population health characteristics. On a global scale, the estimated incidence of preeclampsia ranges between 2% and 8% of all pregnancies, but the burden is disproportionately higher in low- and middle-income countries, where access to prenatal care and early detection is limited [37]. In many developing nations, preeclampsia and eclampsia contribute to a substantial proportion of maternal and perinatal mortality, reflecting both diagnostic and therapeutic disparities.

Geographic and Socioeconomic Variations

In high-income countries, improvements in antenatal surveillance, maternal education, and healthcare infrastructure have contributed to a gradual decline in severe cases and related mortality [38]. However, in sub-Saharan Africa, South Asia, and parts of Latin America, preeclampsia continues to account for a significant percentage of maternal deaths. These differences are largely driven by variations in healthcare accessibility, delayed recognition of symptoms, and limited implementation of preventive strategies such as LDA prophylaxis [39]. Environmental stressors, nutritional deficiencies, and higher rates of untreated hypertension further amplify risk in resource-constrained regions [40].

Racial and Ethnic Disparities

Epidemiologic studies consistently show that preeclampsia incidence is not uniform across racial and ethnic groups [40]. Women of African and South Asian descent are generally at greater risk compared to the Caucasian population, even after adjusting for socioeconomic and clinical factors. Genetic predisposition, differing patterns of inflammation and vascular response, and unequal access to healthcare all contribute to these disparities [39]. Additionally, women from marginalized communities may experience cumulative social and environmental stressors that elevate the risk of hypertensive disorders during pregnancy [41].

Maternal and Clinical Risk Factors

Several maternal characteristics increase the likelihood of developing preeclampsia [42]. Chronic hypertension and pre-existing cardiovascular disease remain the most significant contributors [31]. Metabolic conditions such as obesity, insulin resistance, and diabetes mellitus also heighten susceptibility due to underlying endothelial dysfunction [43]. Autoimmune disorders, including systemic lupus erythematosus and antiphospholipid syndrome, further elevate risk through abnormal immune activation and vascular injury [42].

Advanced maternal age (above 35 years) and very young maternal age (below 18 years) are additional demographic risk factors, often compounded by comorbidities or limited prenatal care [44,45]. Multiple gestation pregnancies, in which placental mass and hormonal output are increased, are associated with higher rates of preeclampsia [46,47]. A positive family history or previous occurrence of the condition strongly predicts recurrence, suggesting a heritable component influenced by both maternal and paternal genes [48].

Lifestyle and Environmental Contributors

Lifestyle factors, including poor diet, limited physical activity, and exposure to environmental toxins such as air pollution or heavy metals, have also been implicated in the pathogenesis of preeclampsia. Nutritional deficiencies - particularly in calcium, vitamin D, and antioxidants - may exacerbate oxidative stress and endothelial dysfunction [49]. Smoking, while paradoxically associated with a lower incidence in some studies, poses numerous other risks and should never be considered protective.

Public health implications

Recognizing the global differences in preeclampsia prevalence highlights the need for tailored, context-specific prevention strategies. Strengthening screening programs, conducting early risk assessments, and implementing LDA prophylaxis among high-risk populations can significantly reduce the disease burden. Narrowing the gap between high- and low-resource settings requires not only clinical interventions but also public health initiatives focused on nutrition, education, and equitable access to maternal healthcare services.

Future directions and research gaps

Although progress has been made in preventing and detecting preeclampsia early, important knowledge gaps remain that require further study. One major focus for future research is the development of personalized prophylactic strategies based on maternal biomarkers and individual risk profiles. Current uniform approaches, such as offering LDA to all high-risk women, may not fully consider biological differences in drug metabolism, vascular response, or inflammatory pathways [50]. Therefore, future investigations should emphasize biomarker-guided stratification models that incorporate genomic, proteomic, and metabolomic data to improve the prediction of disease susceptibility.

Emerging evidence also supports the use of advanced screening techniques, including maternal serum markers, uterine artery Doppler velocimetry, and machine learning-based prediction algorithms, to identify women at the highest risk during the first trimester [51]. These technologies could allow for earlier initiation of prophylactic therapy, potentially improving outcomes. However, further validation and cost-effectiveness analyses are needed before routine clinical implementation. Another important area for exploration is the personalization of aspirin dosing. Studies suggest that factors such as body weight, genetic polymorphisms, and metabolic variability may influence aspirin efficacy [52]. Tailoring the dose to individual pharmacokinetic and pharmacogenomic profiles could enhance preventive effectiveness while minimizing adverse effects [53].

Finally, there remains a critical need to understand the long-term cardiovascular consequences of preeclampsia for mothers. As preeclampsia is now recognized as an early marker of future cardiovascular disease, longitudinal studies are essential to determine how early interventions-such as aspirin prophylaxis or lifestyle modifications-might modify this risk trajectory.

Overall, future research should move toward a precision medicine framework, integrating clinical, genetic, and environmental data to guide prevention, improve risk prediction, and inform postpartum surveillance strategies.

Clinical practice-related challenges

Despite clear evidence supporting aspirin prophylaxis in high-risk pregnancies, translating research into clinical practice remains challenging. Poor adherence is one of the most significant barriers-many women discontinue therapy due to forgetfulness, gastrointestinal discomfort, or uncertainty about safety. Addressing these issues through patient education, counseling, and simplified dosing regimens is crucial.

Delayed initiation of therapy is a frequent challenge. Aspirin prophylaxis is most effective when started before 16 weeks of gestation, but late identification of risk factors or delayed antenatal visits often results in suboptimal timing [54,55]. Enhancing screening systems and raising awareness among primary care providers are crucial to promoting the timely initiation of therapy.

In low-resource settings, access to risk screening tools and medications may be limited, and competing health priorities can hinder implementation. Strategies such as community-based screening, integration into existing maternal health programs, and subsidized medication access could improve equity in prevention efforts. Table 1 summarizes the key future directions, research gaps, and clinical challenges in preeclampsia prevention, highlighting current limitations in practice, proposed research solutions, and their potential implications for individualized care, early detection, and long-term maternal health outcomes.

**Table 1:** Future directions, research gaps, and clinical challenges in preeclampsia prevention* ^*^Data synthesized from references [50–55] This table summarizes current limitations in individualized prophylaxis, early screening, aspirin dosing, and long-term maternal health, as well as proposed research directions and their implications for clinical practice AI: artificial intelligence

Focus area/challenge	Current limitations/underlying issues	Future research directions/potential solutions	Expected impact/implications for practice
Individualized prophylaxis	Uniform aspirin protocols do not consider biological variability among women	Develop biomarker- and risk-based stratification models incorporating genomic, proteomic, and metabolomic data	Improved prediction accuracy and tailored prophylactic interventions
Early screening	Risk identification often occurs after 16 weeks of gestation	Evaluate combined first-trimester screening with maternal serum markers, uterine artery Doppler, and AI-based models. Enhance early screening and improve awareness among healthcare providers	Enables earlier initiation of preventive therapy and better maternal-fetal outcomes. Timely initiation before 16 weeks to maximize benefits
Personalized aspirin dosing	One-dose-fits-all approach ignores body weight, genetic, and metabolic differences	Explore dose adjustments based on pharmacogenomics and pharmacokinetic profiles	Enhanced aspirin efficacy with minimized side effects
Long-term maternal health	Limited understanding of post-preeclampsia cardiovascular risks	Conduct longitudinal studies assessing cardiovascular outcomes and the benefits of early interventions	Improved long-term care and preventive strategies for women with a history of preeclampsia
Precision medicine framework	Fragmented data and inconsistent risk prediction tools	Integrate clinical, genetic, and environmental factors into a unified precision-medicine model	More accurate, individualized prevention and follow-up care
Poor adherence	Non-compliance due to forgetfulness, side effects, or lack of understanding	Strengthen patient counseling, simplify dosing, and emphasize benefits during antenatal visits	Higher adherence and improved preventive efficacy
Limited access in low-resource settings	Shortage of screening tools and inconsistent drug availability	Integrate screening into community health programs; ensure affordable or subsidized aspirin supply	Greater equity in preventive care and reduced disease burden
Inconsistent clinical guidelines	Conflicting recommendations on dose, timing, and eligibility criteria	Harmonize guidelines and strengthen professional training	Improved clinician confidence and standardized care delivery

## Conclusions

LDA has become a cornerstone in the prevention of PE, offering a simple, safe, and cost-effective strategy to reduce maternal and perinatal morbidity. Its mechanism of action - targeting platelet aggregation, inflammation, and endothelial dysfunction - addresses key pathways in the disease’s pathogenesis. The greatest benefit is observed when therapy is initiated before 16 weeks of gestation, at daily doses ranging from 60 to 150 mg, most commonly 75 mg. Despite its proven efficacy, widespread implementation remains limited due to inconsistent screening practices, uncertainty about optimal dosing, and variable clinical adherence. Future research should focus on precision-based approaches, including biomarker and genetic profiling, to personalize prophylactic strategies. Improved clinician education, standardized guidelines, and broader public health integration are essential to ensure equitable access, particularly in low-resource settings. By translating existing evidence into consistent clinical practice, LDA can continue to play a pivotal role in improving maternal and fetal outcomes and mitigating the global burden of PE.
